# A study of association between early menarche and anxiety in undergraduate 
 students

**DOI:** 10.12688/f1000research.149757.1

**Published:** 2024-07-17

**Authors:** Poorva Bakshi, Sharanya B. Shetty, Abira Sharma, Vasudha K G, Keshava Pai, Radhika K, Priyanka Renita D'Souza, Reshma N S

**Affiliations:** 1 Kasturba Medical College, Mangalore, Manipal Academy of Higher Education, Manipal, Karnataka, 576104, India; 2 Department of Psychiatry, Kasturba Medical College, Mangalore, Manipal Academy of Higher Education, Manipal, Karnataka, 576104, India

**Keywords:** early menarche, anxiety, young adults, mental health in young, early puberty

## Abstract

**Background:**

Anxiety has been reported to be one of the most common epidemics in recent years. The present study focused on understanding the association between early menarche and the prevalence of anxiety and anxiety symptoms among adult undergraduate students.

**Methods:**

This was an observational, case-control study. The sample included 146 young female adults aged more than or equal to 18 years pursuing the Bachelor of Medicine and Bachelor of Surgery (MBBS) and Bachelor of Dental Sciences (BDS). Using an online questionnaire, participants were asked to recall and enter the age at which they attained menarche. We used the Generalized Anxiety Disorder 7- Item Questionnaire (GAD-7) to measure the severity of their present anxiety symptoms.

**Results:**

The results showed a significant increase in anxiety symptoms in participants who had early menarche compared to those who did not have early menarche. The mean score on the GAD-7 Questionnaire for the cases was 9.93 and the control group was 6.89. The GAD-7 scores among the cases group were significantly higher in the GAD-7 scores than in the control group.

**Conclusions:**

This study concluded that early menarche is associated with higher anxiety levels in young adults.

## Introduction

Anxiety is the world’s most common mental health disorder, affecting 301 million people worldwide in 2019. It has also been reported that women are affected more by anxiety disorders than men, and their symptoms surge during childhood or adolescence. It is also stated that approximately one in four people with anxiety disorders receive treatment, which reflects that only a minority of the population affected is receiving adequate healthcare. ^1^

The United Nations Sustainable Development Goal (UN SDG 3) focuses on good health, which includes mental health, whereas the UN SDG 5 focuses on gender equality. An intersection between these two goals is the mental health of women, which has long been neglected, leading to a gap in the quality of the healthcare provided to men and women. To bridge this gap, it is imperative to study the factors affecting mental health specific to women. ^2^

In recent decades, there has been a trend of decreasing mean age at menarche in India. ^3^ Early menarche is defined as menarche at or below 12 years of age. ^4^ Eating habits and a higher body mass index have been implicated as risk factors for early menarche. ^4^

Puberty is a formative period during which several hormonal, reproductive, and social changes occur. The psychological effects of early puberty may not be limited to adolescence but may also affect mental health during adulthood. ^5^

A study conducted on secondary school female students aged 12 to 17 years reflected the association between menarche and the prevalence of anxiety and depression comorbidities. ^6^

Studies have evaluated the difference in the effect of pubertal timing between males and females. While early puberty tends to have a positive impact in males, it is linked to increased symptoms of depression among females. ^7^

Social stress after puberty and changes in peer, sexual and family connections have been linked to emotional issues in early adolescence. ^7^^,^^8^

The long-term developmental consequences of pubertal timing reported that girls who mature early continue to have a higher risk of anxiety throughout adolescence and early adulthood. ^9^^,^^10^ Because early menarche leads to an abrupt transition, it leaves little time for emotional preparation. Girls who mature early may experience anxiety and related symptoms. ^11^

Several studies have reported an association between early menarche and psychosocial pathologies in adolescents. Early menarche is associated with increased substance abuse, elevated depression, and suicidal behaviors in adolescent girls. ^12^^,^^13^

The effect of early menarche on mental health in adulthood remains controversial. Some studies have shown that early menarche is associated with depressive symptoms and antisocial behavior in adults, ^14^ while other studies have shown that there is no association between early menarche and symptoms of depression. ^15^ An association has been found between anxiety disorders in adult women and early perceived pubertal timing. ^16^

The fluctuation of estrogen levels during the reproductive lifespan of women has been frequently studied and linked to various psychiatric disorders. ^17^ However, the role of estrogen in anxiety has not been determined. Increased anxiety levels are observed during menarche, which is the time at which estrogen levels become elevated. ^6^ In contrast, animal experiments have shown that acute and chronic estrogen administration have anxiolytic effects. ^18^ Estrogen acts through three estrogen receptor (ER) subtypes, which have different effects on anxiety. Estrogen Receptor alpha (ER alpha) has anxiogenic effects, estrogen receptor beta (ER beta) has anxiolytic effects, and G protein-coupled estrogen receptor (GPR30) has both anxiolytic and anxiogenic effects. ^19^

### Rationale for the study

According to studies that have been conducted in this area, there is an established correlation between early puberty and mental illness in the adolescent population. ^9^^–^^13^ However, the association between early menarche and psychiatric illnesses in adulthood is not well understood. ^14^^–^^16^ We aimed to understand whether there is an association between early puberty and anxiety symptoms in women aged more than or equal to 18 years. Age at menarche was chosen for this study because it is an objective measurement. We selected undergraduate students pursuing Bachelor of Medicine and Bachelor of Surgery (MBBS) and Bachelor of Dental Sciences (BDS), as it was presumed that academic stressors among these students may be similar, thus reducing an important confounding factor.

### Aims

This research aimed to understand the relation between early menarche and anxiety during adulthood.

## Methods

**Study setting:** The study was conducted in Kasturba Medical College and Hospital, Mangalore.

**Study design:** This study was conducted as an analytical observational case control study.

**Study participants:** Female undergraduate students studying in Kasturba Medical College, Mangalore and Manipal College of Dental Sciences, Mangalore.

### Inclusion criteria

Cases:

1. Age more than or equal to 18 years
2. Age at menarche less than or equal to 12 years
3. Undergraduate students willing to consent for the study

Controls:

1. Age more than or equal to 18 years
2. Age at menarche greater than 12 years
3. Undergraduate students willing to consent for the study

### Exclusion criteria

Cases and controls

1. Age less than 18 years

3. Participants unwilling to give consent

4. Incomplete filling of questionnaire

5. Participants who could not recall age at menarche

**Study duration:** The study was conducted over 7 months, from June 2023 to December 2023.

### Sample size

The estimated sample size for the study was 76 for each group, with 5% significance level and 80% power. The total sample size estimated for the study was 152 participants.

$$n={\left({\text{Z1}}_{\alpha }+{\text{Z1}}_{\beta }\right)}^{2}\;\sigma^{2}/d^{2}$$

$$n={\left(1.96+0.84\right)}^{2}\;{\left(31.1\right)}^{2}/{\left(10\right)}^{2}$$

n = 75.8

Z1 _α_ = 1.96 is a standard normal value for 95% confidence interval

Z1 _β_ = 0.84 is a standard normal value at 80% power

σ = standard deviation = 31.1

d = clinically significant difference = 10

**Sampling method:** Convenient sampling method

### Methodology

The study questionnaire was uploaded and shared online on Google Forms. A link to the electronic questionnaire was distributed in the college campus via e-mail, WhatsApp, and Online class groups. The questionnaire had an introductory page describing the background and aims of the study, ethical information for the participants, and informed consent. Once the participant clicked on the ‘I agree’ button, they were directed to the next sections of the questionnaire. Participants were able to leave the questionnaire at any stage before the submission process. If they left in between, their responses were not saved. Responses were saved only by clicking on the ‘submit’ button. By completing the questionnaire, participants acknowledged their voluntary consent to be included in the study.

### Tool for data collection

The questionnaire had the following sections.

Section A: General participant information

This contains the sociodemographic information of the participants, including age, place of residence, and so on.

Section B: Age at menarche and menstrual history

This consists of age at menarche based on recall, history of dysmenorrhea, any known thyroid disorder, and any known gynecological disorder.

Section C: Generalized Anxiety Disorder 7-Item (GAD-7) Questionnaire ^20^

The GAD-7 Scale is a seven-point scale commonly used to assess anxiety in clinical as well as educational settings. Participants marked their answers on a scale of not at all, several days, more than half the days, and nearly every day. The GAD-7 score is calculated by assigning scores of 0 to “not at all,” 1 to “several days,” 2 to “more than half the days,” 3 to “nearly every day.” Thus, the scores range from 0 to 21. The interpretation of the score would be 0–4, minimal anxiety; 5–9, mild anxiety; 10–14, moderate anxiety; and 15–21, severe anxiety.

Section D: Anxiety symptoms

In this section, further questions were asked regarding the duration and treatment of anxiety symptoms experienced by the participants.

**Biological materials required**: none.

### Data analysis

Statistical analysis was performed using IBM SPSS Statistics for Windows version 29 (IBM Corp., Armonk, N.Y., USA). ^21^ A copyright licence was obtained by the institution.

Statistical significance was set at p value<0.05. Descriptive statistics such as mean, proportions, and standard deviation were used to express the results. The t-test was used for continuous variables, and the chi-square test was used for categorical variables.

## Results

The present study comprised a total of 146 participants after excluding participants who did not adhere to our inclusion and exclusion criteria.

The age of the participants in our study ranged from 18 to 25 years. The mean age was 20.54±1.7 years.

The age at menarche of the participants ranged from 10 to 16 years. The mean age at menarche of our participants was 12.48±1.17 years.

The participants were grouped into case and control groups based on age at menarche. The participants with age at menarche less than or equal to 12 years were the cases and the control group consisted of participants with age at menarche greater than 12 years. We had 74 participants in the cases group and 72 participants in the control group.

The mean score for the cases on the GAD-7 questionnaire was 9.93 (Standard deviation=6.05). The mean score for the controls on the GAD-7 questionnaire was 6.89 (Standard deviation=5.24). An independent t-test was performed, and it was found that the GAD-7 scores of the cases were significantly higher than the GAD-7 scores of the controls with a (t(144)=3.26, p<0.05). Thus, the difference in the GAD-7 scores between the cases and controls was found to be significant.

Among the cases, 20.3% had minimal anxiety, 32.4% had mild anxiety, 21.6% had moderate anxiety, and 25.7% had severe anxiety. Among the controls 38.9%, 30.6%, 25.0% and 5.6% had minimal, mild, moderate, and severe anxiety, respectively.

In the cases, 66 of 74 participants entered their age at the onset of anxiety symptoms. Among the controls, 56 out of 72 participants entered their age at the onset of anxiety symptoms. These findings are summarized in Table 1.

**Table 1.** Age of onset of anxiety symptoms among cases and controls.

Age of onset of anxiety symptoms	Cases				Controls			
	Frequency	Percent (%)	Valid percent (%)	Cumulative percent (%)	Frequency	Percent (%)	Valid percent (%)	Cumulative percent (%)
Less than 10 years of age	4	6.1%	6.1%	6.1%	1	1.8%	1.8%	1.8%
Within 10-13 years of age	5	7.6%	7.6%	13.7%	3	5.4%	5.4%	7.2%
Within 14-17 years of age	18	27.2%	27.2%	40.9%	14	25%	25%	32.2%
More than 18 years of age	39	59.1%	59.1%	100%	38	67.8%	67.8%	100%
Total	66				56			

Among the cases out of 74 participants, 65 responded to the question regarding the duration of their anxiety symptoms. Among the controls, 55 out of 72 participants responded to questions regarding the duration of their anxiety symptoms. The findings regarding the duration of the anxiety symptoms are summarized in Table 2.

**Table 2.** Duration of anxiety symptoms among cases and controls.

Duration of anxiety symptoms	Cases				Controls			
	Frequency	Percent (%)	Valid percent (%)	Cumulative percent (%)	Frequency	Percent (%)	Valid percent (%)	Cumulative percent (%)
Less than 6 months	20	30.8%	30.8%	30.8%	18	32.7%	32.7%	32.7%
6 months to 1 year	11	16.9%	16.9%	47.7%	16	29.1%	29.1%	61.8%
More than 1 year	34	52.3%	52.3%	100%	21	38.2%	38.2%	100%
Total	65				55			

Of the 146 participants in the present study, 122 answered the question regarding whether they had received treatment for their anxiety symptoms. 20.5% had received treatment for their symptoms. Thirty participants reported details of the treatment they received. Of these, 12 participants (40%) received pharmacological treatment, 16 (53.3%) received psychological treatment, 1 (3.3%) received both psychiatric and Ayurvedic treatment, and 1 (3.3%) received crystal stone treatment.

Among 146 study participants, 26 participants reported to have known gynecological condition, among them 23 reported to have polycystic ovarian syndrome. Six participants reported to have known thyroid condition.

## Discussion

The present study focused on the association between early menarche and anxiety during adulthood. This was an analytical observational case-control study of female undergraduate students aged more than or equal to 18 years.

The mean age at menarche in our study was 12.48±1.17 years. Their ages ranged from 10 to 16 years. The mean age at menarche was found to be lower than the mean age at menarche in India calculated as of 2005 which was 13.76 years. ^3^ More recently, studies have shown a decline in the mean age at menarche between mothers and daughters. ^22^ A study conducted among North Indian girls in 2023 found the mean age of menarche to be 13.13±1.23 years. ^23^ The mean age at menarche in our study was found to be even lower than these.

The present study found that anxiety symptoms were significantly higher in participants with an early age at menarche. These findings further support those of numerous studies, which show that early puberty and menarche are associated with greater rates of morbidity of anxiety and other psychiatric illnesses than relatively late menarche. ^9^^–^^13^ Other studies have found that females who mature early are more likely to experience symptoms of psychiatric illnesses, including anxiety, throughout adolescence and during the early stages of adulthood. ^9^^,^^10^

Emotional problems in early adolescence have been connected to social stress following puberty and modifications in peer, sexual, and family relationships. ^7^^,^^8^ It is evident that in females, menarche becomes a sudden transition that provides little time for emotional preparation and can cause anxiety and related symptoms. ^11^ During the perimenarcheal phase, participants described feeling puzzled, worried, and ambivalent, but eventually acclimated to these changes. ^11^

These findings can also be explained by estrogen variations in women. Several studies have attempted to explain the effects of estrogen on mental health. ^24^^–^^26^ Menarche, the period when estrogen levels rise, is associated with higher anxiety levels. ^17^ However, the anxiolytic effects of both acute and chronic estrogen treatments have been demonstrated in animal models. ^18^ Estrogen has distinct effects on each subtype of the receptor through a complex interaction. ^19^ This study followed an objective metric, age at menarche, to investigate the relationship between anxiety during adulthood and early puberty.

Among the cases, 20.3% had minimal anxiety, 32.4% had mild anxiety, 21.6% had moderate anxiety, and 25.7% had severe anxiety. Among the controls 38.9%, 30.6%, 25.0% and 5.6% had minimal, mild, moderate, and severe anxiety, respectively. A larger percentage of the women in the case group experienced severe anxiety. However, a greater percentage of the women in the control group experienced minimal anxiety.

In our study, we found that 59.1% of cases and 67.9% of controls first experienced anxiety symptoms after 18 years of age. In contrast, 27.3% of the cases and 25% of the controls first experienced anxiety symptoms between the ages of 14-17 years of age. A previous study found that the mean age of onset of generalized anxiety disorder was between 21.1 and 34.9 years. ^27^ However, this was only partially reflected in our study, probably because the mean age of our participants was 20.54±1.7 years.

According to our study, the duration of anxiety symptoms was less than 6 months in 30.8% of cases and 32.7% of controls. The duration of anxiety symptoms was between 6 and 12 months in 16.9% of cases and 29.1% of controls. The duration of anxiety symptoms was greater than one year in 52.3% of the cases and 38.2% of the controls. A study found the mean duration of anxiety symptoms to be 15.2 months, ^28^ which was in line with our findings.

Of the 122 participants who answered the question, 20.5% received treatment for their symptoms. A study by the World Health Organization on the treatment gap in anxiety disorders also showed that only 27.6% of the respondents who had anxiety disorder had received treatment of any form, and only 9.8% had possibly received adequate treatment. It is evident that there is a need to diagnose anxiety disorders and improve the treatment quality. ^29^

Of the participants who reported receiving treatment, 12 participants (40%) received pharmacological treatment, 16 (53.3%) received psychological treatment, 1 (3.3%) received both psychiatric and Ayurvedic treatment, and 1 (3.3%) received crystal stone treatment. In general, the first-line treatment for generalized anxiety is cognitive behavioral therapy (CBT) and pharmacotherapy (SSRIs, SNRIs). Others include benzodiazepines for acute cases, metacognitive therapy, mindfulness techniques, physical activities, and Acceptance and Commitment Therapy). ^30^ Most participants in our study relied on allopathic treatments, whereas a minority resorted to alternative treatment methods.

This study sheds light on anxiety symptoms associated with early menarche. Further implications of this study include understanding other psychiatric comorbidities associated with early menarche and puberty.

### Limitations

Other stressors, genetic predisposition, lifestyle, environmental causes, and medical history, which may be confounding variables, were not considered in this study. A larger sample size should be considered, and a population-based study should be conducted to increase the likelihood of generalizability of the entire population. Future studies with a longitudinal cohort design are warranted.

## Conclusion

This study indicated that individuals with early menarche had significantly higher anxiety symptoms during adulthood. Healthcare providers need to be sensitized to early pubertal timing in young adults with anxiety. It is a matter of concern that early menarche could have a serious negative impact on developing adolescents and young adults.

### Pre-registered data analysis

The data analysis in this study was not pre-registered.

### Ethical considerations

The protocol was approved by the Institutional Ethics Committee Kasturba Medical College,

Mangalore (Reg. No. ECR/541/Inst/KA/2014/RR-20) before the commencement of the study, and Ethical Approval was obtained. Protocol number of the study used was IEC KMC MLR 05/2023/212. The date of approval was 17/05/2023.

## Consent

The introductory form of the online survey had information about the background, aim of the study and the consent form. Once the participant clicked on the ‘I agree’ button, this was considered consent for participating in the study and the participant was taken to the rest of the questionnaire. The participants had the right to withdraw from the study at any time.

## Data Availability

Open Science Framework: A study of association between early menarche and anxiety in undergraduate students. https://doi.org/10.17605/OSF.IO/RA2V6 ^31^ This project contains the following underlying data:
• Association between Early Menarche and Anxiety in Undergraduate Students.pdf (Questionnaire which was circulated via google forms). • Consent Form.docx • Complete Participant Responses. xlsx (record of all responses collected) • STROBE Checklist. Docx Data are available under the terms of the Creative Commons Attribution 4.0 International license (CC-BY 4.0). This study was based on the STROBE (The Strengthening the Reporting of Observational studies in Epidemiology) guidelines. ^32^

## Software availability

Statistical analysis was performed using IBM SPSS Statistics for Windows version 29 (IBM Corp., Armonk, N.Y., USA). ^21^ The copyright license was as follows: obtained from our institution. An open-source alternative is Jamovi version 1.x. ^33^
