# Supplementary material for: A study of association between early menarche and anxiety in undergraduate  students
Source: F1000Res. 2024 Aug 9;13:807. Originally published 2024 Jul 17. [Version 2] doi: 10.12688/f1000research.149757.2 (PMC11336558; doi:10.12688/f1000research.149757.2)
Supplement: Peer review history

# Reviewer report: Reviewer response for version 2

**DOI:** 10.5256/f1000research.170051.r312738

**Published:** 2024-08-20

**Authors:** Gayatri Bhatia

**Affiliations:** 1 All India Institute of Medical Sciences, Rajkot, India

The suggestions have been satisfactorily addressed. The manuscript may be accepted for indexing.

Is the work clearly and accurately presented and does it cite the current literature?

Partly

If applicable, is the statistical analysis and its interpretation appropriate?

Yes

Are all the source data underlying the results available to ensure full reproducibility?

Yes

Is the study design appropriate and is the work technically sound?

Partly

Are the conclusions drawn adequately supported by the results?

Partly

Are sufficient details of methods and analysis provided to allow replication by others?

Yes

Reviewer Expertise:

Psychiatry, addiction, mental health in special populations, occupational health

I confirm that I have read this submission and believe that I have an appropriate level of expertise to confirm that it is of an acceptable scientific standard.

# Reviewer report: Reviewer response for version 1

**DOI:** 10.5256/f1000research.164254.r304526

**Published:** 2024-07-30

**Authors:** Sanjeev Badiger

**Affiliations:** 1 A J Institute of Medical Sciences and Research Centre, Mangaluru, India

The topic selected is novel , but more details could have added regarding matching. Other factors like socioeconomic conditions and environmental factors could have been added to avoid bias but this was mentioned in the limitation of the study 

 Results and discussion part: satisfactory with good number of references 

 Limitations of the study: Mentioned about the limitations

Is the work clearly and accurately presented and does it cite the current literature?

Yes

If applicable, is the statistical analysis and its interpretation appropriate?

Yes

Are all the source data underlying the results available to ensure full reproducibility?

Yes

Is the study design appropriate and is the work technically sound?

Partly

Are the conclusions drawn adequately supported by the results?

Yes

Are sufficient details of methods and analysis provided to allow replication by others?

Partly

Reviewer Expertise:

Public health, Infectious Diseases and non communicable diseases

I confirm that I have read this submission and believe that I have an appropriate level of expertise to confirm that it is of an acceptable scientific standard.

# Response

**Published:** 2024-08-05

**Authors:** Sharanya Shetty

**Affiliations:** Psychiatry, Kasturba Medical College, Mangalore, Manipal Academy of Higher Education, Mangalore, Karnataka, India

Dear Sir,

 Thank you very much for your review of our article. We are pleased to hear that you found our study to be novel. We appreciate your insightful comments and suggestions.

1. Regarding your suggestion for considering factors like socioeconomic conditions and environmental factors for avoiding the bias, we acknowledge the importance of considering these factors and its impact on menarche and anxiety. Though we have mentioned about this in the limitations, we have made an attempt to discuss regarding social, cultural factors, guidance, communication factors in the discussion in addition to the limitations in the revised manuscript. In future research we plan to explore the influence of these variables to provide a more comprehensive understanding.

We appreciate your positive feedback on results and discussion part and the limitations. Once again thank you for your valuable feedback and recognition of our contribution to understanding the association between early menarche and anxiety in undergraduate students. We will consider your suggestions for future research directions.

 Regards,

 Dr. Sharanya B. Shetty

 Department of Psychiatry, Kasturba Medical College, Mangalore, Manipal Academy of Higher Education, Manipal, India

# Reviewer report: Reviewer response for version 1

**DOI:** 10.5256/f1000research.164254.r304527

**Published:** 2024-07-24

**Authors:** Gayatri Bhatia

**Affiliations:** 1 All India Institute of Medical Sciences, Rajkot, India

This study could have been more powerful if controlled for whether formal/informal guidance was received regarding pubertal changes, and support perceived during that time. Also, factors like family environment, temperament, history of traumatic experiences play a significant mediating role in this context. This study needs to address these factors, if not in methodology, atleast in discussion, limitations and future directions.

Is the work clearly and accurately presented and does it cite the current literature?

Partly

If applicable, is the statistical analysis and its interpretation appropriate?

Yes

Are all the source data underlying the results available to ensure full reproducibility?

Yes

Is the study design appropriate and is the work technically sound?

Partly

Are the conclusions drawn adequately supported by the results?

Partly

Are sufficient details of methods and analysis provided to allow replication by others?

Yes

Reviewer Expertise:

Psychiatry, addiction, mental health in special populations, occupational health

I confirm that I have read this submission and believe that I have an appropriate level of expertise to confirm that it is of an acceptable scientific standard, however I have significant reservations, as outlined above.

# Response

**Published:** 2024-08-05

**Authors:** Sharanya Shetty

**Affiliations:** Psychiatry, Kasturba Medical College, Mangalore, Manipal Academy of Higher Education, Mangalore, Karnataka, India

Dear Madam,

 Thank you for your valuable feedback on our manuscript. We appreciate your insightful comments and suggestions, which has undoubtedly contributed to improving the quality of our manuscript. We have tried our best to address your concerns in the modified manuscript.

1. We understand the importance of controlling for formal/informal guidance received regarding pubertal changes and support. Though we had not controlled this in our study, we have carefully considered your suggestions and made revisions to the discussion part and included regarding the importance of guidance, communication and support during puberty.
2. We appreciate your observations of the significant mediating role of traumatic experience that can play a major role. We have incorporated this in discussion highlighting its role in increasing the risk of anxiety. In addition to the above, we have also discussed on the role of social and cultural environment on puberty in the discussion section.
3. The confounding factors like  family environment, temperament, history of traumatic experience, guidance and support during puberty which were not explored in our study has been highlighted in the limitation section. We have also highlighted the need for these to be incorporated in the future studies.

Thank you again for your valuable feedback and we will consider your suggestions for future research directions.

 Regards

 Dr. Sharanya B. Shetty

 Department of Psychiatry, Kasturba Medical College, Mangalore, Manipal Academy of Higher Education, Manipal, India
